# A single-center, retrospective study-spring-evaluating the efficacy and safety of recombinant human vascular endothelial inhibitor combined with anti-PD-1 in elderly patients aged 80 and above with NSCLC

**DOI:** 10.3389/fimmu.2024.1402018

**Published:** 2024-06-24

**Authors:** Tian Xing, Qianqian Gao, Hongbin Zhu, Jianrong Gao, Ganglin Yan

**Affiliations:** ^1^ Chaohu Hospital Affiliated with Anhui Medical University, Chaohu, China; ^2^ Department of Respiratory and Critical Care Medicine, Chaohu Hospital Affiliated with Anhui Medical University, Chaohu, China

**Keywords:** patients aged 80 and above, NSCLC, Recombinant Human Endostatin Injection, endo, anti-PD-1

## Abstract

**Aim:**

To investigate the efficacy and safety of combining Recombinant Human Endostatin Injection (marketed as Endo) with anti-PD-1 in elderly patients aged 80 and above with non-small cell lung cancer (NSCLC).

**Methods:**

Retrospective analysis of 181 patients with NSCLC aged 80 and above treated in the Department of Respiratory and Critical Care Medicine at Chaohu Hospital, affiliated with Anhui Medical University, from June 2019 to January 2024. Patients who received at least one cycle of combined Endo with anti-PD-1 were included based on inclusion criteria. Clinical and pathological data were collected, including complete blood count, liver and kidney function, electrocardiogram, coagulation function, thyroid function, cardiac enzymes, and whole-body imaging. Adverse events were recorded with a final follow-up on January 25, 2024. The primary endpoints were progression-free survival (PFS) and overall survival (OS), with safety as a secondary endpoint.

**Results:**

This study involved 14 elderly patients with NSCLC aged over 80. Median progression-free survival (mPFS) was 102 days, and median overall survival (mOS) was 311 days. Subgroup analyses based on treatment cycles showed a non-significant 441-day mPFS increase in the long-term group (≥6 cycles, 5 patients) compared to the short-term group (<6 cycles, 9 patients). However, the mOS in the long-term group significantly exceeded the short-term group by 141 days, with statistical significance (*P*=0.048). Further categorization revealed a 204-day shorter mPFS in the monotherapy maintenance group (Endo or Immunol) compared to the combination maintenance group (Endo combined with Immunol, 441 days). The mOS of the monotherapy maintenance group was longer (686 days) than the combination maintenance group (311 days), but no statistical significance (*P*= 0.710, 0.920). Throughout the treatment, 77 adverse events were recorded, mainly grade 1–2, with no new treatment-related reactions occurred. Overall, the safety of Endo combined with anti-PD-1 was considered good and manageable.

**Conclusion:**

The combination of Endo and anti-PD-1 could be an effective treatment choice for patients with NSCLC aged 80 and above.

## 1 Introduction

Lung cancer stands as a primary contributor to global cancer-related fatalities (1), with nearly 20% of deaths in China attributed to this disease, surpassing the world average of approximately 18.4% (2, 3). Non-small cell lung cancer (NSCLC) represents the predominant subtype (4), and with increased life expectancy, the incidence among elderly patients with NSCLC is steadily climbing (5). For this demographic, selecting between radiotherapy, chemotherapy, or surgery is intricate, considering factors like the cytotoxic effects of chemotherapy drugs, surgical risks, and the presence of underlying health conditions (6). As a result, elderly patients with NSCLC face a dilemma: a general reluctance toward comprehensive treatment, yet an enduring unmet clinical need.

Immunotherapy, a potent approach that boosts the body’s immune system to combat cancer, has become a pivotal force in the battle against the disease (7). NSCLC stands out as one of the cancers highly responsive to immune checkpoint inhibitors (ICIs), proven to significantly alleviate symptoms and prolong the survival of certain advanced patients with NSCLC (8, 9). Additionally, immunotherapy demonstrates enhanced tolerance and fewer side effects, making it a favorable option for elderly patients with compromised physical conditions (3).

Tumor angiogenesis has been identified as a crucial therapeutic target for malignant tumors. By modulating signaling pathways linked to angiogenesis, effective inhibition of tumor growth can be achieved (10, 11). Multiple studies have shown that combining anti-angiogenic drugs with chemotherapy, targeted therapy, and immunotherapy enhances efficacy in patients with NSCLC (12), providing a moderate extension of their survival (13, 14).

Currently, identifying an effective treatment plan for elderly patients with NSCLC is an urgent clinical imperative, and the potential of combining anti-angiogenic drugs with immunotherapy emerges as a novel therapeutic strategy. As a novel recombinant human vascular endothelial inhibitor, Endo, when paired with a PD-1 inhibitor, markedly suppresses tumor growth in NSCLC mice by improving the tumor microenvironment and activating autophagy (15). Furthermore, the combination of anti-PD-1 with anti-angiogenic drugs shows promise in enhancing the survival rates of advanced NSCLC patients (16). Health authorities have greenlit the gradual integration of this combination therapy into clinical practice. Thus, this study systematically reviews the efficacy and safety of combining Endo with immunotherapy (anti-PD-1) (17) in patients with NSCLC aged 80 and above.

## 2 Materials and methods

### 2.1 Study population

A retrospective analysis was performed on clinical data from patients with NSCLC aged 80 and above who underwent Endo combined with anti-PD-1 at the Department of Respiratory and Critical Care Medicine, Chaohu Hospital, Anhui Medical University, spanning June 2019 to January 2024. The immunosuppressants administered were Camrelizumab (8) and Tislelizumab (9). This study adhered to the Helsinki Declaration and received approval from the Ethics Committee of Chaohu Hospital, Anhui Medical University.

The inclusion criteria were: (1) Age greater than or equal to 80 years; (2) Histologically confirmed NSCLC staged according to the American Joint Committee on Cancer (AJCC) Lung Cancer Staging Criteria (8th edition); (3) Received a minimum of one cycle of Endo combined with anti-PD-1 post-diagnosis. Exclusion criteria included: (1) Insufficient pathological data; (2) Concurrent presence of other malignancies; (3) Underwent radiotherapy, chemotherapy or surgical treatment during the treatment process; (4) Positive driver gene status and received targeted therapy.

### 2.2 Treatment approach

Patients underwent a combination of Endo and anti-PD-1. Throughout the follow-up, surveillance encompassed blood routine, liver and kidney function, electrocardiogram, coagulation function, thyroid function, cardiac enzymes, and imaging examinations. Adverse events were documented until the final follow-up on January 25, 2024.

### 2.3 Efficacy evaluation

Treatment effectiveness was appraised using the Response Evaluation Criteria in Solid Tumors (RECIST) (18). The primary study endpoints include progression-free survival (PFS), measuring the time from the initiation of Endo combined with anti-PD-1 to disease progression, and overall survival (OS), reflecting the time from the initiation of Endo combined with anti-PD-1 to death or the last follow-up. The secondary study endpoint is safety.

### 2.4 Adverse event assessment

All adverse events were defined and graded according to the National Cancer Institute Common Terminology Criteria for Adverse Events (NCI-CTCAE) version 5.0.

### 2.5 Follow-up

The follow-up cutoff date was January 25, 2024. Follow-up was conducted through inpatient and outpatient clinical records, telephone interviews, and the collection of data on patient PFS and OS.

### 2.6 Statistical methods

Statistical analysis was conducted using SPSS 29.0 software. Survival curves were plotted using the Kaplan-Meier method, and the median progression-free survival (mPFS) and median overall survival (mOS) were estimated using the Kaplan-Meier method with a reported 95% confidence interval (CI). Group comparisons were analyzed using the Log-rank test, with statistical significance set at *P* < 0.05.

## 3 Results

### 3.1 Inclusion process

**Figure 1:**
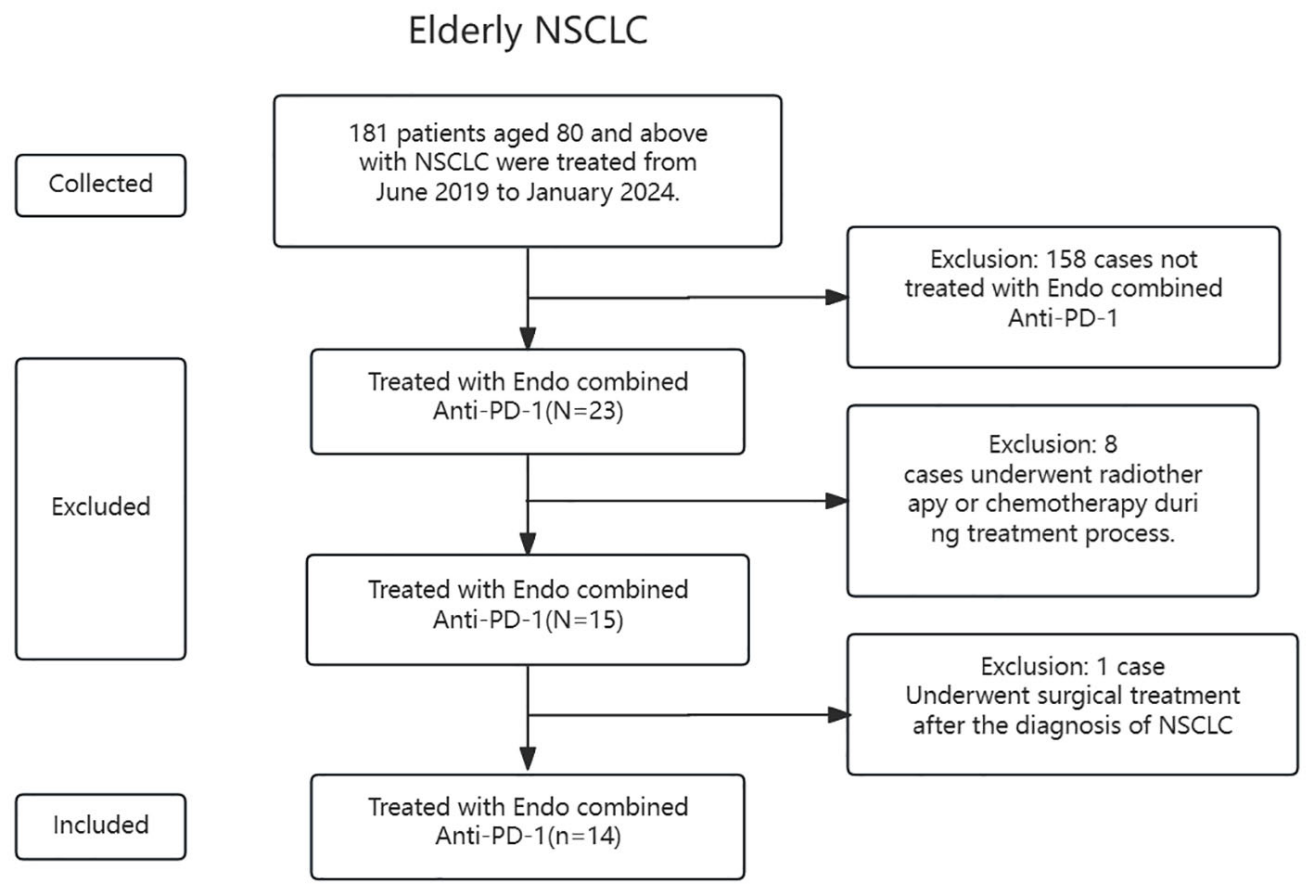
Inclusion process.

A total of 181 patients with NSCLC aged 80 and above, who underwent treatment in the Department of Respiratory and Critical Care Medicine at Chaohu Hospital, Anhui Medical University, from June 2019 to January 2024, were initially collected. Following the inclusion and exclusion criteria established for the study, 158 patients were excluded as they did not receive Endo combined with anti-PD-1. Additionally, eight patients had undergone radiotherapy or chemotherapy during treatment, and one patient had undergone surgical intervention after lung cancer diagnosis. Ultimately, this study included 14 elderly patients with NSCLC aged 80 and above (refer to **Figure 1**).

### 3.2 Clinical and pathological data of all enrolled patients

This study included a total of 14 patients, and their clinical pathological data were analyzed. Among them, 12 patients (86%) were male, and 2 patients (14%) were female. Seven patients (50%) had a history of smoking, while seven patients (50%) had no smoking history. Tumor TNM staging revealed 7 cases (50%) in stage IV, 4 cases (29%) in stage III, 2 cases (14%) in stage II, and 1 case (7%) in stage I. Squamous cell carcinoma accounted for 11 cases (79%), and adenocarcinoma for 3 cases (21%). All 14 patients (100%) had an ECOG score of 2, as shown in **Table 1**.

**Table 1:** Clinical and pathological data of all enrolled patients.

Variables	Cases
Sex	
Male	12(86%)
Female	2(14%)
Smoking	
Yes	7(50%)
No	7(50%)
Pathological type	
Squamous cell carcinoma	11(79%)
Adenocarcinoma	3(21%)
TNM	
I	1(7%)
II	2(14%)
III	4(29%)
IV	7(50%)
ECOG performance status	
Grade 1	0(0%)
Grade 2	14(100%)

### 3.3 PFS and OS of all included patients

**Figure 2:**
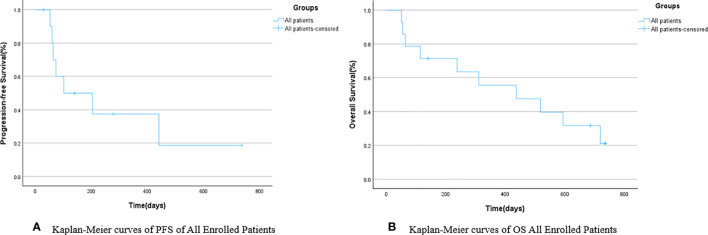
Kaplan-Meier curves of PFS **(A)** and OS **(B)** of all included patients.

Survival follow-up was conducted for all patients until the last follow-up date. Survival analysis was performed for the 14 patients included in the study, and survival curves were plotted. The survival analysis results showed that the mPFS for all patients was 102 days (95% CI: 0.000–281.055), as shown in **Figure 2A**; the mOS was 311 days (95% CI: 0.000–675.849), as shown in **Figure 2B** and **Table 2**.

**Table 2:** mPFS and mOS of all included patients.

	Overall	95%CI	
		Lower Bound	Upper Bound
mPFS	102	0.000	281.055
mOS	311	0.000	675.849

### 3.4 PFS and OS for long and short treatment term groups

**Figure 3:**
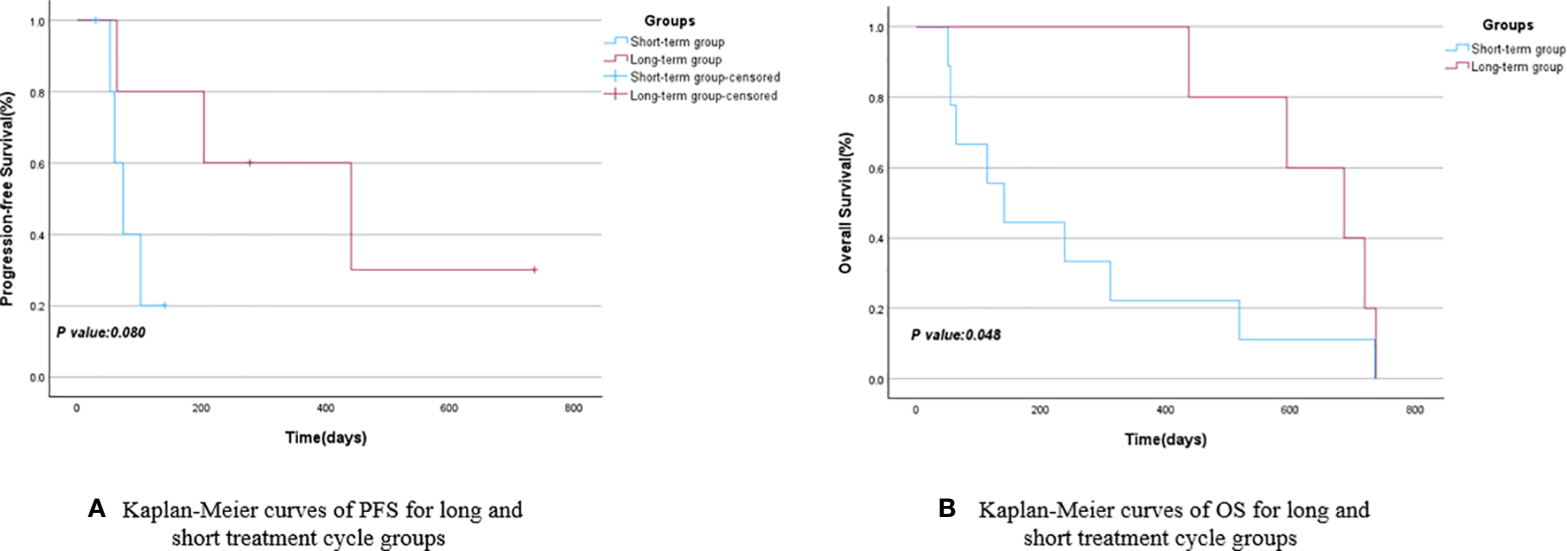
Kaplan-Meier curves of PFS **(A)** and OS **(B)** for long and short treatment cycle groups.

**Figure 4:**
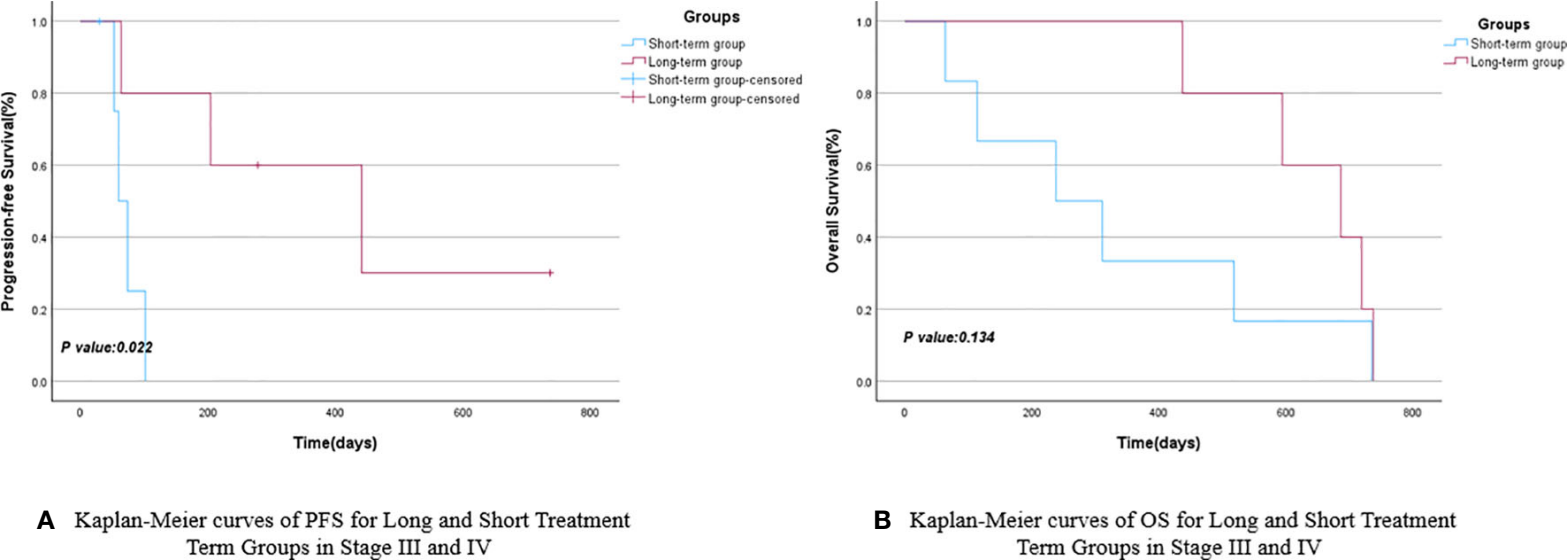
Kaplan-Meier curves of PFS **(A)** and OS **(B)** for long and short treatment cycle groups in stage III and IV.

Patients were grouped based on their treatment cycles, categorized into the long-term group (≥6 cycles) and short-term group (<6 cycles). The mPFS for the long-term group was 441 days (95% CI: 71.325–810.675), while the short-term group had an mPFS of 74 days (95% CI: 43.941–104.059), as depicted in **Figure 3A**. The *P*-value comparing PFS between the two groups showed no statistically significant difference. The mOS for the long-cycle and short-term groups were 686 days (95% CI: 488.469–883.531) and 141 days (95% CI: 62.112–219.888), respectively. The *P*-value comparing OS between the two groups was 0.048, indicating a significant improvement in OS for the long-term group compared to the short-term group, as shown in **Figure 3B** and **Table 3**. A further subgroup survival analysis of patients with stage III and IV NSCLC revealed that the mPFS of the long-cycle group (441 days, 95% CI: 71.325–810.675) was longer than that of the short-cycle group (60 days, 95% CI: 39.420–80.580), with a statistically significant *P*-value (*P* = 0.022). The mOS for the long-cycle and short-cycle groups was 686 days (95% CI: 488.469–883.531) and 238 days (95% CI: 1.551–474.449), respectively, with a *P*-value that was not statistically significant (*P* = 0.134). Refer to **Figure 4** and **Table 4**.

**Table 3:** PFS and OS for long and short treatment cycle groups.

	short-term group	long-term group	95%CI		95%CI		*P* Value
			Lower Bound	Upper Bound	Lower Bound	Upper Bound	
mPFS	74	441	43.941	104.059	71.325	810.675	0.080
mOS	141	686	62.112	219.888	488.469	883.531	0.048

**Table 4:** PFS and OS for long and short treatment cycle groups in stage III and IV.

	short-term group	long-term group	95%CI		95%CI		*P* Value
			Lower Bound	Upper Bound	Lower Bound	Upper Bound	
mPFS	60	441	39.420	80.580	71.325	810.675	0.022
mOS	238	686	1.551	474.449	488.469	883.531	0.134

### 3.5 PFS and OS for monotherapy maintenance group and combination maintenance group

**Figure 5:**
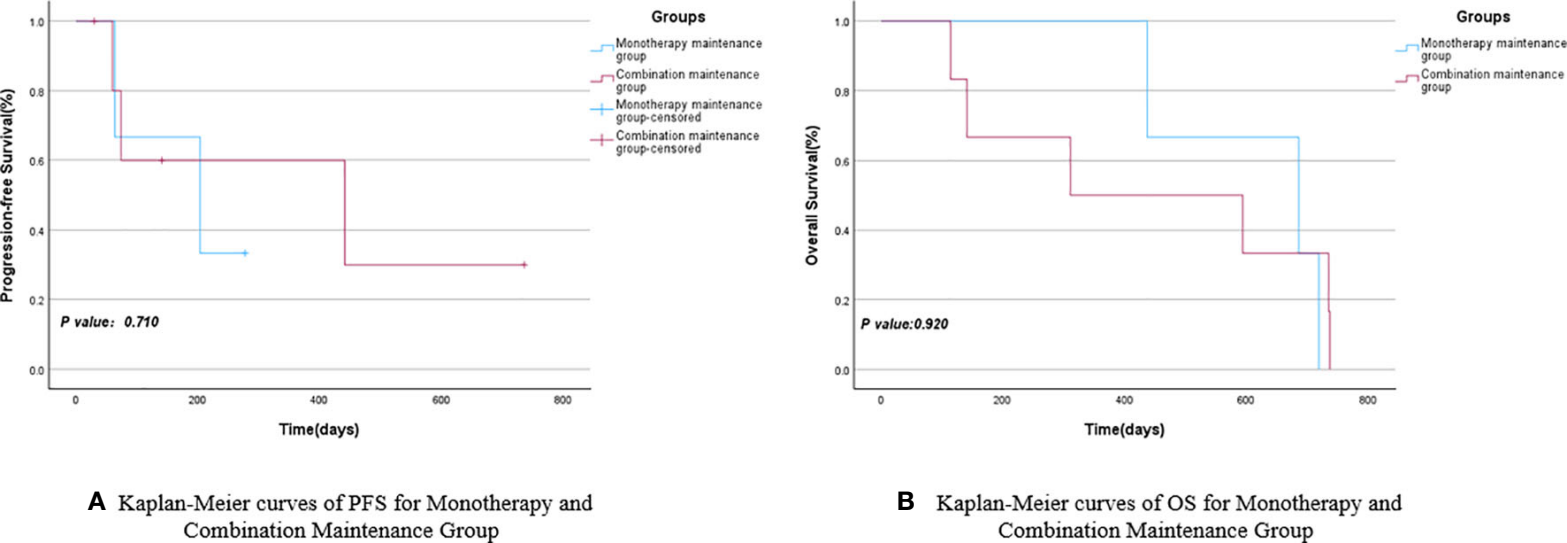
Kaplan-Meier curves of PFS **(A)** and OS **(B)** for Monotherapy and Combination Maintenance Groups.

**Figure 6:**
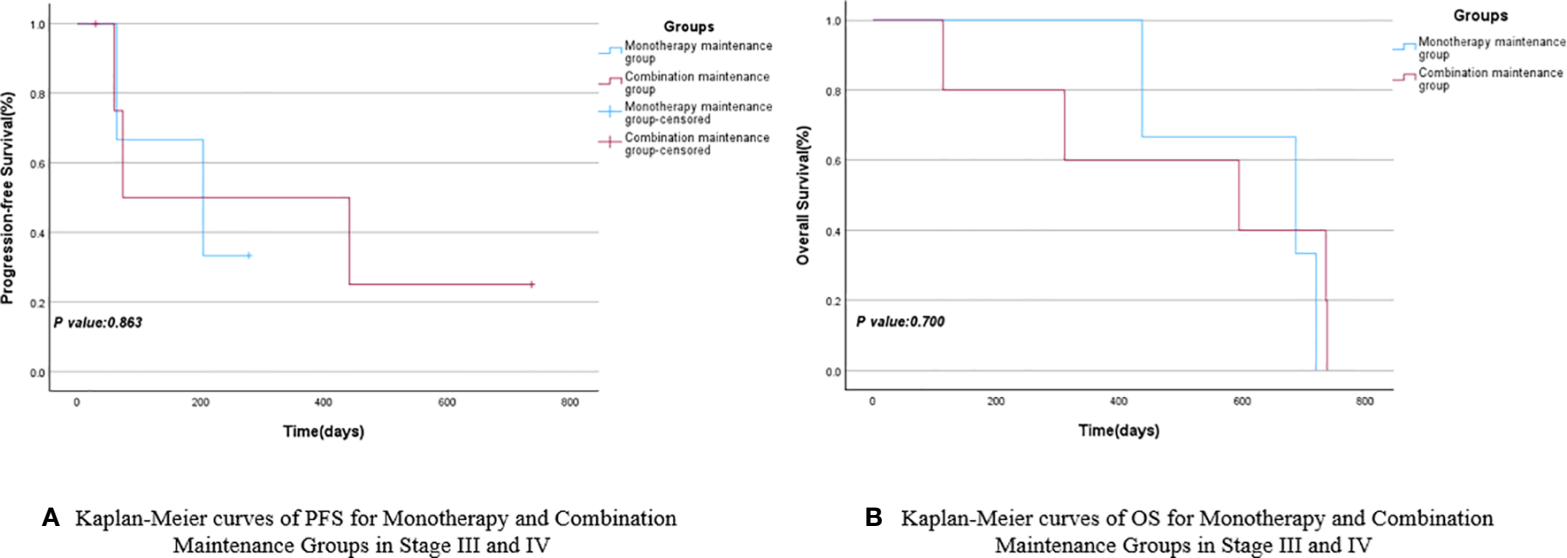
Kaplan-Meier curves of PFS **(A)** and OS **(B)** for Monotherapy and Combination Maintenance Groups in stage III and IV.

Based on the different subsequent maintenance treatment plans for the 14 NSCLC patients included in the study, they were divided into the monotherapy maintenance group (Endo or anti-PD-1) and the combination maintenance group (Endo combined with anti-PD-1). The mPFS for the monotherapy maintenance group was 204 days (95% CI: 0.000–428.047), while the combination maintenance group had an mPFS of 441 days (95% CI: 0.000–1013.451), with a *P*-value of 0.710. The mOS for the combination maintenance group and the monotherapy maintenance group were 311 days (95% CI: 0.000–854.713) and 686 days (95% CI: 287.517–1084.483), respectively, with a *P*-value of 0.920. There were no statistically significant differences in PFS and OS between the monotherapy maintenance group and the combination maintenance group, as shown in **Figure 5** and **Table 5**. Similarly, a survival analysis of patients with stage III and IV NSCLC showed that the mPFS was 204 days (95% CI: 0.000–428.047) in the monotherapy maintenance group, compared to 74 days (95% CI: 0.000–447.380) in the combination maintenance group. The mOS was 686 days (95% CI: 287.517–1084.483) for the monotherapy maintenance group and 594 days (95% CI: 0.000–1201.621) for the combination maintenance group. The *P*-values for mPFS and mOS were not statistically significant (0.863 and 0.700, respectively). See **Figure 6** and **Table 6** for more details.

**Table 5:** PFS and OS for monotherapy and combination maintenance groups.

	Monotherapy Maintenance Group	Combination Maintenance Group	95%CI		95%CI		*P* value
			Lower Bound	Upper Bound	Lower Bound	Upper Bound	
mPFS	204	441	0.000	428.047	0.000	1013.451	0.710
mOS	686	311	287.517	1084.483	0.000	854.713	0.920

**Table 6:** PFS and OS for monotherapy and combination maintenance groups in stage III and IV.

	Monotherapy Maintenance Group	Combination Maintenance Group	95%CI		95%CI		*P* value
			Lower Bound	Upper Bound	Lower Bound	Upper Bound	
mPFS	204	74	0.000	428.047	0.000	447.380	0.863
mOS	686	594	287.517	1084.483	0.000	1201.621	0.700

### 3.6 Adverse reactions

Adverse reactions occurring during the treatment of 14 enrolled patients were recorded, documenting a total of 77 adverse events of grade 1 or higher, including 4 cases with Grade 3–4 adverse reactions. Lymphocyte decrease occurred in 3 cases (21%), and anemia in 1 case (7%). Among the Grade 1–2 adverse reactions, there were 73 cases, with the most common being anemia in 13 cases (93%), hematuria in 57%, leukocyte decrease in 6 cases (43%), bleeding in 6 cases (43%), and proteinuria in 5 cases (36%) as shown in **Table 7**.

**Table 7:** Treatment-related adverse events during the treatment period.

Adverse Events	Cases	Grade 1–2	Grade 3–4	Grade 5
Anemia	14	13(93%)	1(7%)	0(0%)
Thrombocytopenia	3	3(21%)	0(0%)	0(0%)
Leucopenia	6	6(43%)	0(0%)	0(0%)
Neutropenia	2	2(14%)	0(0%)	0(0%)
Lymphocytopenia	5	2(14%)	3(21%)	0(0%)
Hemorrhage	6	6(43%)	0(0%)	0(0%)
Proteinuria	5	5(36%)	0(0%)	0(0%)
Hematuria	8	8(57%)	0(0%)	0(0%)
Aspartate aminotransferase increase	1	1(7%)	0(0%)	0(0%)
Chronic renal disease	1	1(7%)	0(0%)	0(0%)
Creatinine increase	2	2(14%)	0(0%)	0(0%)
Activated partial thromboplastin time prolong	4	4(29%)	0(0%)	0(0%)
Hypercholesterolemia	3	3(21%)	0(0%)	0(0%)
Blood bilirubin increase	1	1(7%)	0(0%)	0(0%)
Hypothyroidism	4	4(29%)	0(0%)	0(0%)
Hypertension	3	3(21%)	0(0%)	0(0%)
Creatine kinase increase	2	2(14%)	0(0%)	0(0%)
First degree atrioventricular block	1	1(7%)	0(0%)	0(0%)
Atrial fibrillation	1	1(7%)	0(0%)	0(0%)
Sinus bradycardia	4	4(29%)	0(0%)	0(0%)
Delirium	1	1(7%)	0(0%)	0(0%)
Total	77	73(95%)	4(5%)	0

## 4 Discussion

Currently, elderly NSCLC patients encounter a dilemma. Platinum-based chemotherapy is frequently employed for advanced NSCLC, but its use often brings about adverse effects such as nausea, vomiting, nephrotoxicity, myelosuppression, and neurotoxicity (19–21). These side effects, coupled with the frail physical condition, low tolerance, and reluctance to undergo treatment, frequently lead elderly patients to discontinue the therapy. Previous studies have affirmed the efficacy and relatively well-controlled safety profile of anti-PD-1 (22) (8, 9) and anti-angiogenic drugs (13–17) in advanced patients with NSCLC. Therefore, this retrospective analysis aims to explore the effectiveness and safety of combining anti-PD-1 with Endo in patients with NSCLC aged 80 and above.

A total of 181 elderly patients with NSCLC aged over 80 were initially enrolled in this study, underscoring the significance and sizable representation of this patient demographic. Unfortunately, during the follow-up period, a considerable number of patients discontinued treatment due to their physical condition and intolerance to the associated toxicities and side effects. This underscores the pressing need in clinical settings for an effective and well-tolerated treatment strategy tailored to meet the unique requirements of this population. Given the known toxic side effects of chemotherapy, alternative non-chemotherapeutic treatment options have been explored. The combination of antiangiogenic drugs with anti-PD-1 represents a novel therapeutic approach widely adopted in clinical practice. This study retrospectively analyzed the efficacy and clinical significance of patients treated with the combination of Endo and anti-PD-1. Ultimately, only 14 cases met the inclusion criteria, with all enrolled patients having an ECOG physical strength status score of 2. Males and squamous carcinomas were prevalent, half of the patients had a history of smoking, and those in stage III and above constituted the majority, comprising 79% of the total patient cohort. The majority of elderly patients with NSCLC presented with intermediate to advanced stages, and half had a history of smoking, possibly linked to less distinctive or more common symptoms (e.g., cough, sputum, hemoptysis). As of the follow-up date on January 25, 2024, survival analysis revealed a mPFS of 102 days and a mOS of 311 days.

Immunotherapy has demonstrated effectiveness in patients with NSCLC (23), with some individuals experiencing sustained responses to immunotherapy-related drugs, leading to the formation of a long-term survivor group (24). To analyze the impact of different durations of maintenance therapy on the survival of elderly patients with NSCLC aged over 80, the enrolled patients were categorized into a long-term group (5 cases) and a short-term group (9 cases) based on their physical condition, treatment readiness, and the duration of subsequent maintenance therapy. The findings revealed that the mPFS and mOS for the long-term group were 441 and 686 days, respectively, while those for the short-term group were 74 and 141 days, respectively. Although the long-term group exhibited longer mPFS and mOS compared to the short-term group, statistical significance was observed only in terms of mOS (*P*<0.05). This underscores the potential benefits of a more extended treatment course for elderly patients with NSCLC, particularly in terms of overall survival. Given that elderly patients in real-world studies often present with later-stage disease due to delayed detection and that this study included relatively few stage I and II patients, we conducted a subgroup analysis of stage III and IV patients. The analysis revealed that both the mPFS and mOS were longer in the long-cycle group compared to the short-cycle group, with a significant difference in mPFS (441 days for the long-cycle group versus 60 days for the short-cycle group). We hypothesize that may be attributed to the ongoing survival of some patients in the combination maintenance group as of the follow-up period, and the overall survival data for these patients may not have matured sufficiently. This study will continue to be monitored, and updates will be provided subsequently.

Furthermore, owing to varying treatment requirements (25, 26) and the emergence of intolerable side effects in some patients, certain participants in the study opted for a single drug (Endo or anti-PD-1) during follow-up maintenance therapy, while others chose to continue the combination therapy. Simultaneously, three enrolled patients did not undergo subsequent maintenance therapy, and two of them only received one subsequent maintenance therapy. These five patients were excluded, and the remaining participants were divided into the monotherapy maintenance group (3 cases) and the combination maintenance group (6 cases) to further investigate whether the efficacy of the combination of anti-angiogenic drugs and anti-PD-1 surpassed that of single-agent maintenance. The survival analyses of the two groups revealed that the mPFS of the monotherapy maintenance group was 204 days, which was shorter than the mPFS of the combination maintenance group (441 days). Conversely, the mOS of the monotherapy maintenance group was 686 days, exceeding the mOS of the combination maintenance group (311 days). Although the monotherapy maintenance group and the combination maintenance group demonstrated contrasting results in terms of mPFS and mOS, none of these findings reached statistical significance (*P*>0.05). In the subsequent survival analysis of maintenance therapy for stage III and IV NSCLC patients, a similar pattern emerged: the mPFS was 204 days in the monotherapy maintenance group compared to 74 days in the combination maintenance group. Similarly, the mOS was 686 days in the monotherapy group and 594 days in the combination group. However, the *P*-values indicated no statistically significant differences between the groups.

Considering the unique characteristics of the elderly NSCLC patient population, we conducted an analysis of the clinicopathological data for 14 enrolled patients. The overall safety profile of Endo combined with anti-PD-1 was found to be favorable. A total of 77 adverse events were documented, with 73 (94.8%) classified as grade 1–2 adverse events and 4 (5.2%) as grade 3–4 adverse events. Notably, there were no grade 5 adverse events. Among these events, anemia (16.9%) emerged as the most common. These findings align with existing studies (27, 28), indicating the absence of new treatment-related adverse events and affirming the overall manageability of the treatment’s safety.

This study, being a single-center retrospective analysis, addresses a substantial demand for treatment among the elderly NSCLC patient population. However, there remains a scarcity of prospective studies considering both patient willingness to undergo treatment and the impact of therapeutic drugs. Additionally, the overall sample size became modest after applying the inclusion criteria, and in subsequent subgroup analyses, while some statistically significant results were obtained, the small sample size and the incomplete maturity of the data at present may pose certain limitations that warrant further exploration. We plan to incrementally update the data in subsequent follow-ups. The primary objective of this study is to investigate whether the combination of Endo and anti-PD-1 represents a novel treatment strategy for elderly patients with NSCLC aged over 80. The current findings suggest advantages of this regimen for the treatment of elderly patients with NSCLC. To further evaluate the clinical value of this treatment approach, we intend to pursue multicenter, prospective studies in the future.

## 5 Conclusions

The combination of Endo and anti-PD-1 could be an effective treatment choice for patients with NSCLC aged 80 and above.

## Data availability statement

The raw data supporting the conclusions of this article will be made available by the authors, without undue reservation.

## Ethics statement

The studies involving humans were approved by Chaohu Hospital Affiliated with Anhui Medical University. The studies were conducted in accordance with the local legislation and institutional requirements. The participants provided their written informed consent to participate in this study.

## Author contributions

TX: Writing – original draft. QG: Writing – original draft. HZ: Writing – review & editing. JG: Writing – review & editing. GY: Writing – review & editing.
